# Neurocomputational mechanisms underlying cross-modal associations and their influence on perceptual decisions

**DOI:** 10.1016/j.neuroimage.2021.118841

**Published:** 2022-02-15

**Authors:** Joshua Bolam, Stephanie C. Boyle, Robin A.A. Ince, Ioannis Delis

**Affiliations:** a School of Biomedical Sciences, University of Leeds UK; b Institute of Neuroscience and Psychology, University of Glasgow UK

**Keywords:** Cross-modal associations, Congruency, Implicit Association Test, EEG, Perceptual decision-making, Hierarchical Drift Diffusion Model

## Abstract

When exposed to complementary features of information across sensory modalities, our brains formulate cross-modal associations between features of stimuli presented separately to multiple modalities. For example, auditory pitch-visual size associations map high-pitch tones with small-size visual objects, and low-pitch tones with large-size visual objects. Preferential, or congruent*,* cross-modal associations have been shown to affect behavioural performance, i.e. choice accuracy and reaction time (RT) across multisensory decision-making paradigms. However, the neural mechanisms underpinning such influences in perceptual decision formation remain unclear. Here, we sought to identify when perceptual improvements from associative congruency emerge in the brain during decision formation. In particular, we asked whether such improvements represent ‘early’ sensory processing benefits, or ‘late’ post-sensory changes in decision dynamics. Using a modified version of the Implicit Association Test (IAT), coupled with electroencephalography (EEG), we measured the neural activity underlying the effect of auditory stimulus-driven pitch-size associations on perceptual decision formation. Behavioural results showed that participants responded significantly faster during trials when auditory pitch was congruent, rather than incongruent, with its associative visual size counterpart. We used multivariate Linear Discriminant Analysis (LDA) to characterise the spatiotemporal dynamics of EEG activity underpinning IAT performance. We found an ‘Early’ component (∼100–110 ms post-stimulus onset) coinciding with the time of maximal discrimination of the auditory stimuli, and a ‘Late’ component (∼330–340 ms post-stimulus onset) underlying IAT performance. To characterise the functional role of these components in decision formation, we incorporated a neurally-informed Hierarchical Drift Diffusion Model (HDDM), revealing that the Late component decreases response caution, requiring less sensory evidence to be accumulated, whereas the Early component increased the duration of sensory-encoding processes for incongruent trials. Overall, our results provide a mechanistic insight into the contribution of ‘early’ sensory processing, as well as ‘late’ post-sensory neural representations of associative congruency to perceptual decision formation.

## Introduction

In everyday life, we encounter situations where we are required to form rapid perceptual decisions based on ambiguous sensory information (Philiastides et al., 2017; Philiastides and Heekeren, 2009). This can involve processing information presented to multiple sensory modalities (Alais et al., 2010; Ghazanfar and Schroeder, 2006), a process commonly referred to as multisensory decision-making (Bizley et al., 2016; Drugowitsch et al., 2014; Franzen et al., 2020; Rapaso et al., 2012). Previous research has shown decision-making benefits deriving from complementary features of information across multiple sensory modalities (Ernst and Bülthoff, 2004). The brain's tendency to systematically map implicitly learnt associations between features of information across sensory modalities is referred to as cross-modal association (Parise and Spence, 2013; Spence and Deroy, 2013; Spence et al., 2011). When exposed to complementary features of sensory information, features that refer to the same object are redundantly associated, forming cross-modal associations, enabling the brain to exploit the correlation between such informational cues when forming perceptual decisions from ambiguous, and often noisy, unisensory information (Bien et al., 2012; Glicksohn and Cohen, 2013) .

Cross-modal associations have been shown to influence the consolidation of multisensory information when forming perceptual decisions (Bizley et al., 2016; Drugowitsch et al., 2014; Engel et al., 2012). This has been evidenced in studies that have used speeded classification paradigms, demonstrating behavioural effects such as increased response speed (i.e. decreased reaction times; RTs; Kayser and Kayser, 2018; Laurienti et al., 2004; Silva et al., 2017), increased choice accuracy (Franzen et al., 2020; Kayser and Kayser, 2018; Kayser et al., 2017; Kim et al., 2008), and improved stimulus detection (Adam and Noppeney, 2014; Aller et al., 2015). These associative influences towards multisensory decision-making are consistently attributed to the modulatory effects of *cross-modal (in)congruency* (Marks, 2004), i.e. modulations in behavioural performance when a multisensory stimulus has two or more features that are (un)favourably mapped. Preferential, or anticipated, cross-modal associations, are referred to as *congruent*, whereas non-preferential, or non-anticipated, cross-modal associations, are referred to as *incongruent*. A paradigmatic example demonstrated extensively across previous literature concerns auditory pitch-visual size associations (Bien et al., 2012; Evans and Tresiman, 2010; Gallace and Spence, 2006; Parise and Spence, 2009; 2008; 2012). Congruent auditory pitch-visual size associations map high-pitch tones with small-size objects, and low-pitch tones with large-size objects, whereas their incongruent counterparts map high-pitch tones with large-size objects, and low-pitch tones with small-size objects.

Previous research has demonstrated that the congruency of auditory pitch-visual size associations modulates behavioural performance, in particular, benefitting the formation of perceptual decisions. For example, Gallace and Spence (2006) found, using a visual discrimination paradigm, that participants responded more rapidly (i.e. decreased RTs) when auditory stimulus pitch (high/low-pitch tones) was congruent with the visual stimulus size (small/large-size disks) than when incongruent or no auditory stimulus was presented. Similarly, Parise and Spence (2008) found that when participants were asked to judge the temporal order of two different-sized visual stimuli (large/small-size grey circles), congruent auditory tones increased choice accuracy (i.e. higher sensitivity temporal order judgements). In contrast, in a follow-up study, participants judged the spatial discrepancy of an auditory stimulus less accurately (i.e. higher just noticeable difference discrimination thresholds) when presented congruently with the visual stimulus, suggesting a decisional bias of congruency and showing that the behavioural effects of congruency depend on the task at hand (Parise and Spence, 2009). Finally, decreased RTs for congruent, compared to incongruent, pairings were found when only one unisensory stimulus feature was presented per trial using an Implicit Association Test (IAT; Parise and Spence, 2012).

The neural basis of cross-modal associations within perceptual decision formation has recently become a focus of human electrophysiology and neuroimaging research (Spence et al., 2011; Bizley et al., 2016). However, the neural mechanisms facilitating these behavioural enhancements remain less well understood. In particular, it is not clear whether such improvements reflect the consequences of ‘early’ sensory processing benefits, or ‘late’ post-sensory changes in decision dynamics, or both. For example, auditory pitch-visual size congruency effects have been identified across two Event-Related Potentials (ERPs) at ∼250 ms and ∼300 ms at parietal and frontal electrodes respectively (Bien et al., 2012), whereas neural modulations of associative semantic congruency have been found in parahippocampal, dorsomedial, and orbitofrontal cortices at ∼100 ms and ∼400 ms post-stimulus (Diaconescu et al., 2011). Similarly, significant differences between congruent and incongruent learned label-object associations have been identified as early as ∼140 ms across occipital regions, whereas mismatches to the learned associations evoked a modulation between ∼340 ms and ∼520 ms across parietal regions (Kovic et al., 2010). Overall, the above studies have started to shed light on the neural underpinnings of associative congruency across various association types, yet they have not provided a conclusive mechanistic account of how the brain uses cross-modal associations to improve the efficiency of perceptual decisions.

Difficulties in identifying the neural basis of cross-modal associations further stems from the utilisation of experimental paradigms that present two or more unisensory features. Previous research has associated multiple neural processes with the observed decision-making benefits, in particular i) multisensory integration; integrating information across sensory modalities into unified percepts (Angelaki et al., 2009; Calvert et al., 2004; Mercier and Cappe, 2020), or ii) a form of selective attention; dividing attentional resources towards attending to task-relevant information in one sensory modality, and ignoring task-irrelevant information in another modality (Bien et al., 2012; Choi et al., 2018; Gallace and Spence, 2006; Marks, 2004). Attending to two simultaneously presented stimulus features may facilitate enhancements to perceptual decision formation from benefits not directly attributed to genuine cross-modal associations. As such, any underlying neural activity will display mixed selectivity representing a variety of sensory, decision-related, and other task-relevant signals (Chandrasekaran, 2017; Dahl et al., 2009; Fusi et al., 2016; Kobak et al., 2016; Park et al., 2014; Raposo et al., 2014; Rigotti et al., 2013). Therefore, it remains difficult to characterise whether cross-modal associations represent early sensory processing benefits and/or late post-sensory changes to decision dynamics during the formation of perceptual decisions.

In this study, we sought to capitalise on the novelty of using a modified variant of the Implicit Association Test (IAT), demonstrated by Parise and Spence (2012), to induce auditory pitch-visual size cross-modal associations from the presentation of one unisensory stimulus feature (i.e. auditory pitch). The IAT presents one stimulus feature per trial and manipulates associative congruency by switching the stimulus feature-response key mappings across blocks of trials. Therefore, the proposed experimental manipulations overcome the methodological limitations present in previous research. First, the presentation of one sensory stimulus feature limits confounding effects from the processes of multisensory integration and selective attention. Second, the manipulation of associative congruency across blocks limits confounding effects from explicit stimulus feature mappings and subjective reporting of cross-modal associations. Thus, by coupling this paradigm with electroencephalography (EEG), we can record the neural activity underlying formulated auditory pitch-visual size associations, which is less likely to be affected by confounding activity attributed to processing multisensory stimuli.

Using this paradigm, we aim to mechanistically characterise the neural dynamics underlying cross-modal associations during perceptual decision formation. To achieve this, we first analysed single-trial EEG activity using multivariate Linear Discriminant Analysis (LDA; Parra et al., 2002; 2005; Philiastides and Sajda, 2006; Philiastides et al., 2006; Philiastides et al., 2014; Sajda et al., 2009). Then, to dissect the constituent processes underlying the effects of pitch-driven associations on perceptual decision formation, we adopted a *neurally-informed cognitive modelling approach* (Delis et al., 2018, Diaz et al., 2017, Franzen et al., 2020, Kayser and Shams, 2015, Turner et al., 2013, Turner et al., 2016, Turner et al., 2017). This approach links underlying latent behavioural variables to hypothesised cognitive processes, and further constrains model fits with recorded neuroimaging data, to interpret the modulation of neural activity under different experimental conditions. Previous neurally-informed cognitive modelling research has provided mechanistic characterisations of neural activity underlying perceptual decision formation, and recently, multisensory decision-making (Delis et al., 2018; Franzen et al., 2020; Mercier and Cappe, 2020). Here we used a neurally-informed Hierarchical Drift Diffusion Model (HDDM; Wiecki et al., 2013) to understand how the neural representations of auditory-driven pitch-size associations drive behavioural benefits to perceptual decision formation. Using this approach, we can extract sensory and decision-specific processes from brain activity and relate these to associative congruency benefits when forming perceptual decisions.

## Methods and materials

### Participants

20 participants (male = 7, female = 13; age range = 19–32 years) were recruited for this study. All participants reported normal/corrected-to-normal vision and normal hearing. Participants were recruited using the University of Glasgow Subject Pool and received £6/hour (UK Sterling) for their participation. The study was approved by the ethics committee of the College of Science and Engineering at the University of Glasgow (CSE application number 300,130,001), and was conducted in accordance with the Declaration of Helsinki.

### Stimuli

We used two auditory and two visual stimuli, which were created and presented using MATLAB (Mathworks) and the Psychophysics Toolbox Extensions (Brainard, 1997). Auditory stimuli consisted of two 300 ms pure tones (‘high’ and ‘low’ pitch, 2000 Hz and 100 Hz respectively). Visual stimuli consisted of two light grey circles (‘small’ and ‘large’, 2 cm and 5 cm, 1.1° and 2.8° of visual angle respectively). The sound intensity of each tone was matched to 72 dB(A) SPL for left and right ears using a sound level metre. Auditory stimuli were presented using Sennheiser headphones and visual stimuli were presented on a Hansol 2100A CRT monitor at a refresh rate of 85 Hz.

### Implicit Association Test

The IAT is a two-alternative forced-choice (2AFC) task that measures implicit perceptual associations between two arbitrary stimulus features by manipulating stimulus feature-response key mappings (Greenwald et al., 1998). In one block of trials, two stimulus features are assigned, or *mapped*, to the same response key, whereas in a separate block of trials, they are assigned to different response keys. Reaction times (RTs) and choice accuracy are collected as dependant variable measurements of behavioural performance (and perceptual choice formation). The IAT assumes that the *congruency* of stimulus feature-response key mappings modulates behavioural performance, with perceptual choices faster (i.e. lower RTs) and more accurate (i.e. higher choice accuracy) when stimulus features are assigned to the same response key than when assigned to different response keys.

**Fig. 1:**
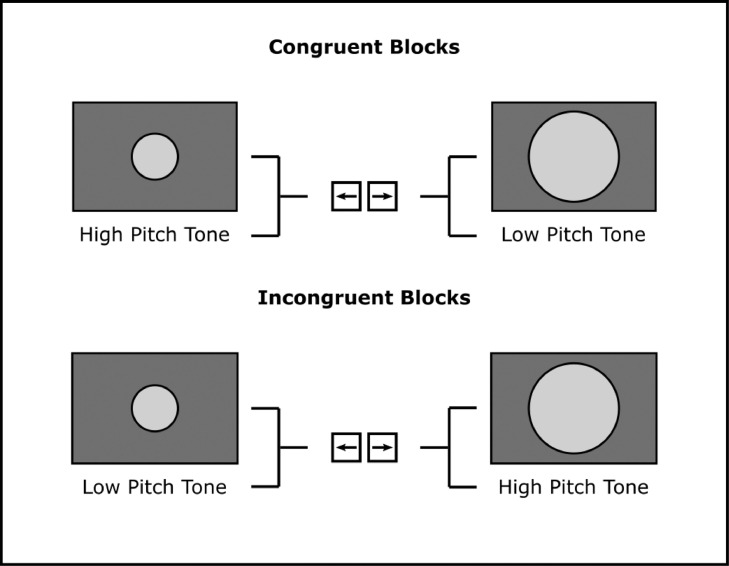
**Implicit association test.** Participants were presented with one unisensory stimulus feature (auditory high/low-pitch tone; visual small/large-size circle) per trial, and were asked to categorize which stimulus feature, within that modality, was presented as quickly and accurately as possible, using the correct response key (left/right). Auditory congruency (congruent/incongruent) was manipulated by switching the stimulus feature-response key mappings across blocks (*top*, congruent block mappings; *bottom*, incongruent block mappings).

This study used a modified version of the IAT, adapted from Parise and Spence (2012), to formulate auditory pitch-visual size cross-modal associations (Fig. 1). In this version, on each block, one auditory (high-pitch/low-pitch tone) and one visual (small-size/large-size circle) stimulus feature are assigned to each response key. Participants are then instructed to categorise as quickly and as accurately as possible which stimulus feature was presented on a single-trial basis using the correctly assigned response key. Congruency was manipulated by switching the stimulus feature-response key mappings across blocks of trials. Congruent mappings assigned high-pitch tones and small-size circles to the left response key, and low-pitch tones and large-size circles to the right response key (Fig. 1, *top*). Incongruent mappings, however, switched the auditory stimulus feature-response key mappings only, so that high-pitch tones and low-pitch tones were assigned to the right and left response keys respectively (Fig. 1, *bottom*). These mappings justify previous findings, which suggested that high-pitch tones are often preferentially associated with small-size visual objects, and vice versa (Gallace and Spence, 2006; Evans and Treisman, 2010; Parise and Spence, 2012). The assigned visual stimulus features remained fixed across blocks for two reasons: (1) Pilot testing found that participants started to exhibit cross-modal associations between visual size and their assigned response keys, rather than their auditory pitch counterparts. Specifically, small-size and large-size visual objects were associated with left and right response keys respectively. (2) In total, experimental sessions ran for ∼3 h (∼2 h for EEG setup/cleanup, ∼1.5 h for the task of 1280 trials per subject). Taken together, this made it difficult to design a cross-modal association experiment where the auditory and visual stimuli were counterbalanced, and participants were not asked to spend more than three hours in a single laboratory session. For these reasons, we chose to only manipulate auditory pitch-response key mappings, therefore manipulating auditory stimulus feature congruency, across blocks. These stimulus feature-response key mapping manipulations are consistent with the mapping manipulations used in the study by Parise and Spence (2012).

### Procedure

Participants completed the experiment in a dark and electrically shielded room. Each block began with instructions on the auditory pitch-visual size mapping between stimuli and response keys (see *Implicit Association Test* section). Participants were given as much time as they needed to memorize the instructions for the upcoming block. Each trial started with a fixation cross presented centrally on-screen for a randomized period (uniform distribution from 500 to 1000 ms). Then, one of the four stimuli (see *Stimuli* section) were selected randomly and presented for 300 ms. Participants were instructed to categorize, as quickly and as accurately as possible, the presented stimulus using the left and right keyboard response keys, as defined by the instructions given for that specific block (see *Implicit Association Test* section). Feedback was given after each trial, with green fixation crosses given for correct response choices, and red fixation crosses given for incorrect response choices. Feedback was provided for a randomised duration (uniform distribution from 300 ms to 600 ms). In total, participants completed 8 blocks (4 blocks each for the congruency of stimulus feature-response key mappings presented in a randomized order) for a total of 1280 trials (160 trials per block; 40 trials for each stimulus feature).

### Analysis of behavioural data

For each participant, median RTs and choice accuracy (calculated as the proportion of correct choices over all trials) were used as dependant variable measurements of behavioural performance. These were calculated separately for two independent variables: i) stimulus feature (auditory: high-pitch/low-pitch tones; visual: small-size/large-size circles), and ii) congruency of stimulus feature-response key mappings (congruent/incongruent). To further assess the effect of switching auditory stimulus feature-response key mappings, we calculated RTs for correct and incorrect choice responses. Trials with RTs less than 300 ms, or more than 1200 ms, were excluded from further analysis, as behavioural performance on such trials is often attributed to “fast guesses” (<300 ms) or attentional lapses (>1200 ms) during testing (Whelan, 2008). Overall, 905 trials (auditory: 677 trials; visual: 228 trials) were excluded from further analysis, leaving a total of 9850 trials for auditory stimuli and 10,323 trials for visual stimuli

As anticipated, RT data was not normally distributed (Whelan, 2008). Therefore, we statistically analysed median RTs and choice accuracy using Wilcoxon Matched-Pairs Signed-Rank tests, and further analysed RTs for correct and incorrect choices responses using Mann-Whitney U Paired tests. Effect sizes were calculated by dividing the Wilcoxon Signed-Rank test statistic (*Z*) by the square root of the test population (*N* = 20) for stimulus features (auditory: high-pitch/low-pitch tones; visual: small-size/large-size circles) and congruency (congruent/incongruent) respectively (Rosenthal et al., 1994). For Mann-Whitney U Paired testing analysing RTs of correct and incorrect choices within each congruency and stimulus feature condition for auditory stimuli, effect sizes were calculated by dividing the Mann-Whitney U test statistic (*Z*) by the square root of the total number of trials for correct and incorrect responses in each congruency condition (congruent/incongruent) and stimulus feature condition (high-pitch/low-pitch tones). For Mann-Whitney U Paired testing analysing RTs of correct and incorrect choices across congruency and auditory stimulus feature conditions, effect sizes were calculated by dividing the Mann-Whitney U test statistic (*Z*) by the square root of the total number of trials in each accuracy condition (correct/incorrect) across congruency and auditory stimulus feature conditions This enabled us to analyse the effects of both congruent/incongruent stimulus feature-response key mapping conditions, and high-pitch/low-pitch tone presentations for correct and incorrect RTs respectively. *Post hoc* power analyses were conducted using G*Power (Faul et al., 2007; 2009) to assess whether any identified significant results were of sufficient statistical power (Cohen 1992a; 1992b; see the *Power analyses* section in *Supplementary materials*). Statistical analysis of all behavioural data was completed using R.

### EEG recording and preprocessing

Continuous EEG data was recorded in a sound-attenuated and electrostatically shielded room using a 128-channel BioSemi amplifier system and ActiView recording software (Biosemi, Amsterdam, Netherlands). Signals were sampled and digitized at 512 Hz, then band-pass filtered online between 0.16 and 100 Hz. Signals originating from ocular muscles were recorded from four additional electrooculography (EOG) electrodes placed below and at the outer canthi of each eye.

Individual blocks of data were preprocessed using the Fieldtrip Toolbox **(**Oostenveld et al., 2011), which was implemented in MATLAB using custom scripts. Epochs of 2 s, from −0.5 to 1.5 s relative to stimulus onset, were extracted and filtered between 0.5 and 90 Hz using a Butterworth filter, before being down-sampled to 200 Hz. Potential signal artefacts were removed using Independent Component Analysis (ICA), using the Fieldtrip toolbox (Oostenveld et al., 2011). Components related to typical eye movement activities, such as blinks, or noisy electrode channels were removed. Horizontal, vertical, and radial EOG signals were further processed using established procedures (Hipp and Siegel, 2013; Keren et al., 2010) and trials with high correlations between eye movements (e.g. saccades) and components in the EEG data removed. Remaining trials with amplitudes that exceeded ±120 µV were also removed. Successful cleaning was verified by visual inspection of single trials.

### EEG signal analysis - Linear Discriminant Analysis

We applied single-trial multivariate Linear Discriminant Analysis (LDA; Parra et al., 2002;^2005^**;** Philiastides and Sajda, 2006; Philiastides et al., 2006; Philiastides et al., 2014; Sajda et al., 2009) to extract EEG components discriminating between congruent and incongruent trials for auditory stimulus-locked EEG data only. Specifically, for a pre-defined time window of interest, this method applies a linear multivariate classifier to EEG data in order to estimate a spatial weighting vector that quantifies the optimal combination of EEG sensor linear weights. When applied to multichannel EEG data, this yields a one-dimensional projection that maximally discriminates between two conditions of interest. This projection represents the ‘discriminating component’ that integrates all signal information across the multichannel EEG array, while reducing effects common to both conditions. Compared to univariate trial-averaging approaches, notably Event-Related/Evoked Response Potential (ERP) analyses, multivariate approaches are better able to spatially integrate information across the multidimensional EEG sensor space, yielding components which both preserve inter-trial signal variability and increase the signal-to-noise ratio (Sajda et al., 2011) for preserved task-relevant information. Note that the term ‘component’ is preferred instead of ‘source’ in order to make clear that this is a projection of all EEG activity correlated with the underlying source.

We used a sliding window approach (Parra et al., 2005; Sajda et al., 2009) to identify a projection of the multichannel EEG signal, $x_{i}\left(t\right)$, where i=[1…Ntrials], and N is the total number of trials, within short time windows that maximally discriminated between congruent and incongruent trials for auditory stimulus features only. All time windows had a width of 50 ms, with the window centre t shifted from −100 ms to 800 ms, relative to auditory stimulus-onset, in 5 ms increments. Specifically, we used logistic regression (Parra et al., 2002; 2005) to learn a 128-channel spatial weighting vector w(t) that achieved maximal discrimination within each time window. This yields a one dimensional projection, $y_{i}\left(t\right)$, for each trial i and given window t:

$$y\left(t\right)=w^{T}x\left(t\right)=\;\sum_{i=1}^{D}w_{i}x_{i}\left(t\right)$$

Here, D represents the number of channels in the multichannel EEG array and T refers to a matrix transpose operator. Our classifier was designed to map component amplitudes, $y_{i}\left(t\right)$, for congruent and incongruent trials, that separates activity maximizing differences and minimizing similarities of effects from neural processes common to both conditions. In discriminating the two congruency categories, the classifier maps negative and positive discriminant component amplitudes to congruent and incongruent trials respectively. Thus, larger negative values indicate a higher likelihood of categorizing auditory stimuli within congruent stimulus feature-response key mappings, and larger positive values indicate a higher likelihood of categorizing auditory stimuli within incongruent stimulus feature-response key mappings, with values near zero reflecting less discriminative component amplitudes.

We quantified classification performance of our classifier for each time window using the area under a receiver operating characteristic (ROC) curve (Green and Swets, 1966), referred to as an $A_{z}$ value, using a leave-one-out cross-validation procedure (Gherman and Philiastides, 2015; Philiastides and Sajda, 2006). To determine group significance thresholds for discriminator performance, we implemented a permutation test, whereby congruent and incongruent trial labels were randomized and submitted to the leave-one-out procedure. This randomization procedure was repeated 1000 times, producing a probability distribution for $A_{z}$, which we used as reference to estimate the $A_{z}$ value leading to a significance level of p<0.05.

Finally, the linearity of our model allowed us to compute scalp projections of the discriminating components resulting from Eq. (1) by estimating a forward model as:

$$a\left(t\right)=\;\frac{x\left(t\right)y\left(t\right)}{y{\left(t\right)}^{T}y\left(t\right)}$$

where the EEG data (x) and discriminating components (y) are organized as matrix and vector notations, respectively, for convenience. Here, the EEG matrix, $x_{i}\left(t\right)$, denotes channel activity across rows and trials across columns for all 5 ms increments in time window t, whereas discriminating components, $y_{i}\left(t\right)$, are organized as single-trial vectors, y(t), with each row is from trial i. Such forward model implementations can be displayed as scalp topographies and interpreted as the coupling between discriminating component amplitudes and observed multichannel EEG activity, whereby vector a(t) reflects the coupling of the discriminating component y(t) that explains most of the activity in x(t), with maps illustrating this optimal component-activity coupling (Philiastides et al., 2014).

### Hierarchical Drift Diffusion Model – description

We fit participants’ behavioural performance i.e. RTs and choice accuracy, with a Hierarchical Drift Diffusion Model (HDDM; Wiecki et al., 2013). Similar to the traditional Drift Diffusion Model (DDM; Ratcliff et al., 2015; R. 2016; Forstmann et al., 2016; Ratcliff and McKoon, 2008; Ratcliff, 1978), the HDDM assumes sensory evidence is stochastically accumulated over time, towards one of two decision boundaries, corresponding to two choice alternatives (e.g. correct or incorrect choices; left or right response keys). For each decisional process, the HDDM returns parameter estimates of four internal components of perceptual decision-making, (1) the rate of evidence accumulation (drift rate), (2) possible *a priori* bias towards one of the two choice alternatives (starting point), (3) the distance between two decision boundaries controlling the amount of evidence required for one particular choice alternative (decision boundary), and (4) the duration of non-decisional processes, which can include time taken for stimulus encoding and motor-response production latency (non-decision time).

### Hierarchical Drift Diffusion Model – fitting

To fit HDDM to participants’ performance and estimate internal decisional processes, we used the HDDM toolbox (Wiecki et al., 2013), an open-source software package, written in Python, that permits custom fits of HDDM variants to participants’ RTs and choice accuracy. The HDDM uses a Bayesian hierarchical framework to estimate the above four parameters, whereby sampled prior probability distributions of the model parameters are updated based on a likelihood function, formed from the data given to the model, to yield posterior probability distributions. The HDDM uses Markov-Chain Monte Carlo sampling within this framework, whereby prior distributions of estimated parameters are iteratively adjusted by a likelihood function that maximizes the log likelihood of predicted mean RTs and choice accuracy (Gamerman and Lopes, 2006). The use of Bayesian hierarchical frameworks, and specifically the HDDM, allows for several benefits relative to traditional (non-hierarchical) DDM analysis. First, such frameworks assume that participants’ samples in a dataset are randomly drawn from a group (Vadekerchkove et al., 2011), thereby constraining participant- and group-level posterior distributions, which yield more stable parameter estimates for individual participants (Wiecki et al., 2013). Second, the HDDM has been found to be more robust in achieving stable parameter estimates in datasets with low numbers of trials, compared to non-hierarchical DDM approaches (Ratcliff and Childers, 2015). Third, rather than quantifying the most likely value for each parameter, uncertainty can be directly conveyed with posterior distributions for each estimated parameter (Wiecki et al., 2013; Navarro and Fuss, 2009; Gelman, 2003). Fourth, and most importantly for our analysis, the HDDM framework supports the use of external variables as regressors of estimated model parameters, to assess the relations between specific parameters with further behavioural or neuroimaging data (Delis et al., 2018; Frank et al., 2015; Franzen et al., 2020; Mercier and Cappe, 2020; Tremel and Wheeler, 2015).

To implement the HDDM, we used a process referred to as ‘accuracy-coding’ (Wiecki et al., 2013), which fits the HDDM to RT distributions that assume the upper and lower decision boundaries corresponding to correct and incorrect choices respectively. We sampled parameter estimates for drift rate (δ), decision boundary (θ), and non-decision time (τ). Starting point (z) was set as the midpoint between the two decision boundaries, since the IAT had no *a priori* bias towards either choice alternative (i.e. response key; Philiastides et al., 2011). We did not include any inter-trial variability parameters in our models as previous studies have shown that it is difficult to achieve stable posterior estimates, particularly with fewer trials (Boehm et al., 2018; Ratcliff and Childers, 2015). For each model, we ran 5 separate Markov chains with 11,000 samples each. For each chain, the first 1000 were discarded as “burn-in”, and the rest subsampled (“thinned”) by a factor of two, to reduce the autocorrelation within and between Markov chains. This is a conventional approach to MCMC sampling, whereby initial samples in the “burn-in” period are based on the selection of a random starting point, and neighbouring samples likely to be highly correlated. Both issues are likely to provide unreliable posterior distributions for estimated parameters. This left 25,000 remaining samples for our model, which constituted the probability distributions for each estimated parameter, allowing us to compute individual parameter estimates for participants and condition categories. To ensure Markov chain convergence, we computed Gelman-Rubin Ȓ statistics between chains (Gelman and Rubin, 1992). This compares within-chain and between-chain variance of estimated parameters both for individual participants and group conditions. We verified that all Ȓ statistics fell between 0.98 and 1.02, which suggests reliable convergence between chains.

### Hierarchical Drift Diffusion Model – EEG regressors

We sought to use our EEG discrimination analysis results to inform the fitting of the HDDM to our behavioural data (i.e. RTs and choices). Specifically, we used the HDDM toolbox (Wiecki et al., 2013) to construct regressors that assessed the trial-by-trial linear relationship between our single-trial EEG discriminator amplitudes (for congruent and incongruent trials) and posterior estimates for drift rate (δ), decision boundary (θ), and non-decision time (τ). In line with our behavioural results, in which we reported a significant effect of RTs decreasing for congruent trials, we hypothesized that component amplitudes would be predictive of increases in the rate of evidence accumulation (drift rate) and decreases in evidence required for categorising auditory stimuli (decision boundary). For the duration of non-decisional processes (non-decision time), we hypothesized that either a) component amplitudes for congruent trials would be predictive of decreases in the duration of non-decisional processes, or b) component amplitudes for incongruent trials would be predictive of increases in the duration of non-decisional processes. Therefore, as part of the model fitting within the HDDM framework, we used our single-trial EEG discriminator amplitudes for congruent and incongruent trials to construct regressors for drift rate (δ), decision boundary (θ), and non-decision time (τ) as follows:

$$\delta =\;\alpha_{0}+\;\alpha_{1}*\left|y_{early}^{max}\right|+\;\alpha_{2}*\;\left|y_{late}^{max}\right|$$

$$\theta =\;\beta_{0}+\;\beta_{1}*\left|y_{early}^{max}\right|+\;\beta_{2}*\;\left|y_{late}^{max}\right|$$

$$\tau =\;\gamma_{0}+\;\gamma_{1}*\;\left|y_{early}^{max}\right|+\;\gamma_{2}*\;\left|y_{late}^{max}\right|$$

where $|y_{early}^{max}|$ and $|y_{late}^{max}|$ are the maximum, single-trial, discriminator amplitudes of subject-specific, stimulus-locked EEG components capturing the highest classification performance between congruent and incongruent trials (corresponding to group peak $A_{z}$ values; Early ∼ 110 ms; Late ∼ 340 ms; see Fig. 3). Coefficients $\alpha_{1},\;\beta_{1},\;\gamma_{1}$ and $\alpha_{2},\;\beta_{2},\;\gamma_{2}$ weight the slope of each parameter by the absolute values of $|y_{early}^{max}|$ and $|y_{late}^{max}|$ respectively, with intercepts $\alpha_{0},\;\beta_{0},\;\gamma_{0}$, on a trial-by-trial basis for each subject and congruency condition. Note that we used the absolute values of our single-trial EEG discriminator amplitudes to construct regressors, since congruent trials were predominantly categorised by negative $|y_{early}^{max}|$ and $|y_{late}^{max}|$ values, and incongruent trials were predominantly categorised by positive $|y_{early}^{max}|$ and $|y_{late}^{max}|$ values respectively (see *EEG signal analysis – Linear Discriminant Analysis* section). Hence, by using these regression coefficients, we were able to assess the trial-by-trial modulatory effects of each identified component on drift rate, decision boundary, and non-decision time in both congruency conditions. Consequently, we can characterise the behavioural benefits of cross-modal associative congruency on perceptual decision formation, dissecting which decisional processes best predict decreases in choice RT.

To assess the posterior predictive power of our regression coefficients, we first calculated the posterior probability densities of samples that differed from 0 using the built-in functions of the HDDM toolbox (Wiecki et al., 2013) corresponding to our pre-defined hypotheses predicting the effect of decreased RTs for congruent trials, and decreased RTs for incorrect responses for congruent trials (albeit not significantly affecting choice accuracy, see *Behavioural results* section). For drift rate and incongruent non-decision time regression coefficients, probability densities were calculated from the proportion of samples greater than 0 (P(δ > 0); P(τ > 0)), whereas for decision boundary and congruent non-decision time regression coefficients, probability densities were calculated from the proportion of samples less than 0 (P(θ < 0); P(τ < 0)). Then, we calculated each coefficient's posterior log-odds by applying the logit function to the proportion of posterior samples in favour of their corresponding hypothesis (Ince et al., 2020). This Bayesian Inference approach was utilised because Bayesian hierarchical modelling frameworks violate the assumption of independence in its posterior estimation sampling procedure, since group-level and participant-level parameter posteriors are simultaneously estimated (Wiecki et al., 2013). Therefore, null-hypothesis significance testing approaches commonly utilised in frequentist approaches to statistical analysis are not recommended. To determine the prevalence of true positive results, implicating strong predictive effects of our regression coefficients on posterior parameter estimations, we further calculated the log posterior odds proportion of a hypothetical sample corresponding to a false-positive rate of ɑ = 0.05 (i.e. a 95% true-positive threshold). Regression coefficient log-odds proportions greater than the hypothetical log-odds proportion of our false positive rate (which is equal to 2.944) suggests highly predictive effects of our regression coefficients on changes to estimated posterior parameters favoured by our hypotheses.

## Results

### Behavioural results

**Fig. 2:**
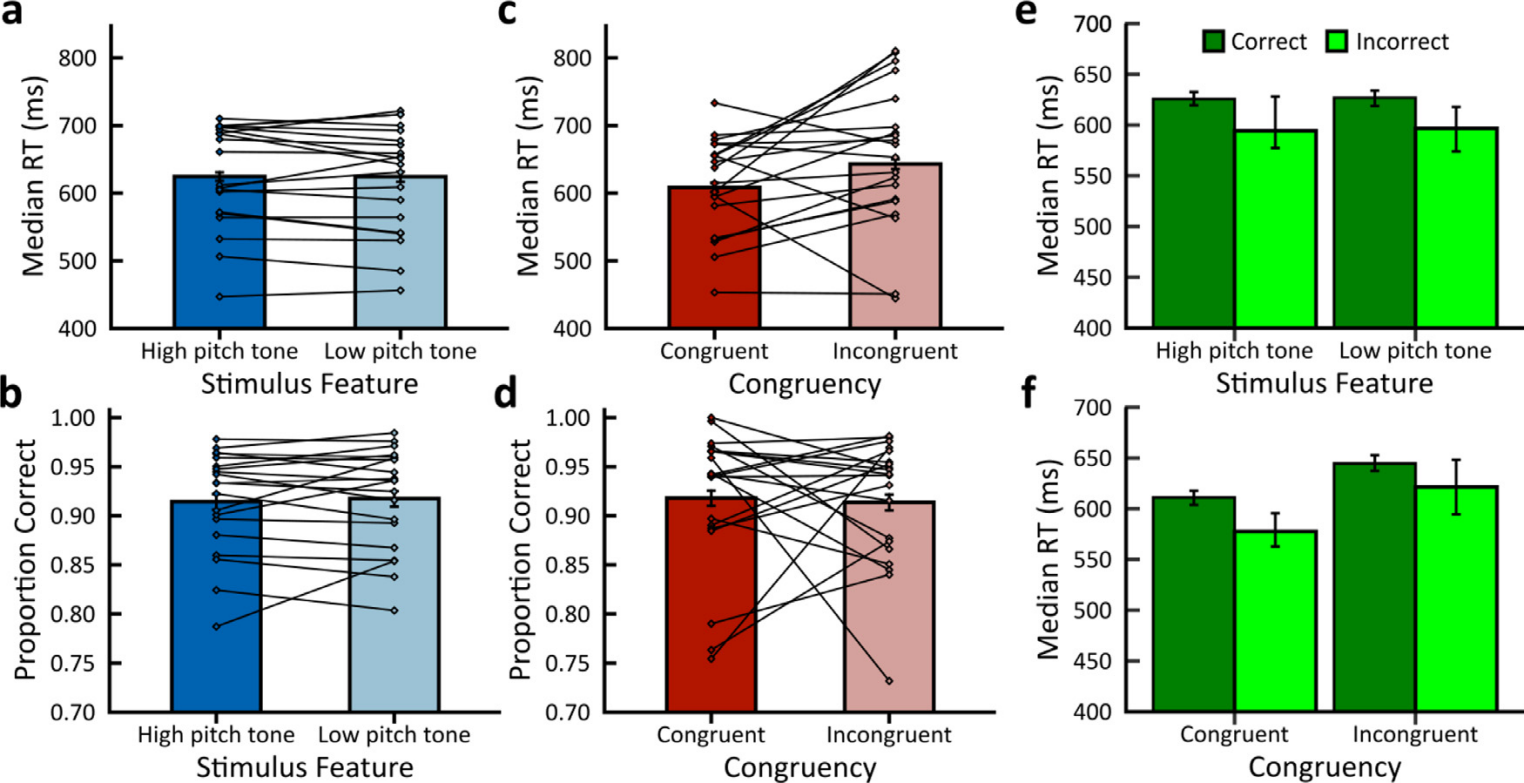
**Behavioural performance.***Left.* Median RTs and choice accuracy (proportion of correct responses) for condition (bars) and participants (scatter points) for **a, b** Stimulus Feature (high/low-pitch tones) and **c, d** Congruency (congruent/incongruent). *Right.* Median RTs for correct and incorrect RT for **e** Stimulus Feature (high/low-pitch tones) and **f** Congruency (congruent/incongruent). For all graphs, 95% Confidence Intervals (CIs) were computed using 1000 bootstrapping random sampling iterations to estimate the distribution of average performance measurements.

Participants responded faster in auditory trials with congruent compared to incongruent stimulus feature-response key mappings (Fig. 2b, Congruent: median = 608 ms post-stimulus offset; Incongruent: 643 ms post-stimulus offset). Wilcoxon Signed-Rank Testing determined this finding to be statistically significant (*Z* = −2.135, *p* = 0.033, effect size = −0.477, Wilcoxon Signed-Rank Testing). This result held for both correct (Fig. 2f, Congruent/Correct: median = 611 ms post-stimulus offset; Incongruent/Correct: median = 645 ms post-stimulus offset, *Z* = −6.940, *p* < 0.001, effect size = −0.073, Mann-Whitney U testing) and incorrect trials separately (Fig. 2f, Congruent/Incorrect: median = 578 ms post-stimulus offset; Incongruent/Incorrect: median = 621 ms post-stimulus offset, *Z* = −2.628, *p* = 0.004, effect size = −0.091, Mann-Whitney U testing). Furthermore, RTs were significantly longer for correct compared to incorrect responses for congruent stimulus feature-response key mappings (Fig. 2f, Correct: median = 611 ms post-stimulus offset; Incorrect: median = 578 ms post-stimulus offset, *Z* = −2.142, *p* = 0.016, effect size = −0.030, Mann-Whitney U Testing), but not for incongruent stimulus feature-response key mappings (Fig. 2f, Correct: median = 645 ms post-stimulus offset; Incorrect: median = 621 ms post-stimulus offset, *Z* = −0.664, *p* = 0.253, effect size = −0.010, Mann-Whitney U Testing). We found no significant effect of stimulus feature on median RTs (Fig. 2a, High-Pitch Tone: median = 625 ms; Low-Pitch Tone: median = 624 ms, *Z* = −0.788, *p* = 0.430, effect size = −0.176, Wilcoxon Signed-Rank Testing). There was also no significant effect when testing correct (Fig. 2e, High-Pitch Tone/Correct: median = 626 ms post-stimulus offset; Low-Pitch Tone/Correct = 626 post-stimulus offset, *Z* = 0.421, *p* = 0.663, effect size = 0.004, Mann-Whitney U Testing) or incorrect trials separately (Fig. 2e, High-Pitch Tone/Incorrect: median = 594 ms post-stimulus offset; Low-Pitch Tone/Incorrect: median = 597 ms post-stimulus offset, *Z* = 0.420, *p* = 0.663, effect size = 0.015, Mann-Whitney U Testing). Furthermore, we found no significant difference in RT between correct and incorrect responses for either high-pitch tones (Fig. 2e, Correct: median = 626 ms post-stimulus offset; Incorrect: median = 594 ms post-stimulus offset, *Z* = −0.994, *p* = 0.172, effect size = −0.013, Mann Whitney-U Testing), or low-pitch tones (Fig. 2e, Correct: median = 627 ms post-stimulus offset; Incorrect: median = 597 ms post-stimulus offset, *Z* = −1.627, *p* = 0.052, effect size = −0.023, Mann Whitney-U Testing).

Regarding choice accuracy, participants had a slightly but not significantly higher proportion of correct responses for auditory trials with congruent compared to incongruent stimulus feature-response key mappings (Fig. 2d, Congruent: proportion correct = 0.918; Incongruent: proportion incorrect = 0.913, *Z* = −0.128, *p* = 0.898, effect size = −0.029, Wilcoxon Signed-Rank Testing). There was also no significant effect of stimulus feature on choice accuracy (Fig. 2b, High-Pitch Tone: proportion correct = 0.914; Low-Pitch Tone: proportion correct = 0.917, *Z* = −0.237, *p* = 0.812, effect size = −0.053, Wilcoxon Signed-Rank Testing).

We found no significant effect of associative congruency on median RTs for visual stimuli (Supplementary Figure 1b, Congruent: median = 581 ms post-stimulus offset; Incongruent: median = 604 ms post-stimulus offset; *Z* = −1.161, *p* = 0.245, effect size = −0.260, Wilcoxon Signed-Rank Testing). We further found no significant effect of visual stimulus feature on median RTs (Supplementary Figure 1a, Small-Size Circle: median = 597 ms post-stimulus offset; Large-Size Circle: median = 586 ms post-stimulus offset, *Z* = −0.863, *p* = 0.388, effect size = −0.193, Wilcoxon Signed-Rank Testing).

Regarding choice accuracy, participants had a slightly, but not significantly, higher proportion of correct responses for trials with congruent compared to incongruent visual stimulus feature-response key mappings (Supplementary Figure 1d, Congruent: proportion correct = 0.957; Incongruent: proportion correct = 0.955, *Z* = −0.055, *p*-value = 0.956, effect size = −0.012. Wilcoxon Signed-Rank Testing). There was also no significant effect of visual stimulus feature on choice accuracy (Supplementary Fig. 1c, Small-Size Circle: proportion correct = 0.954; Large-Size Circle: proportion correct = 0.958; *Z* = −1.161, *p*-value = 0.245, effect-size = −0.260, Wilcoxon Signed-Rank Testing).

To summarize, we found responses for congruent auditory trials were faster than responses for incongruent auditory trials and, in addition, within the set of congruent trials, correct responses were slower than incorrect responses. Furthermore, we found responses for congruent visual trials were not faster nor more accurate compared to incongruent visual trials. Therefore, no significant behavioural improvements as a result of associative congruency were demonstrated when categorising visual stimulus features (see *Supplementary materials* for Figs. 1 and 2 for the behavioural and modelling results for visual stimuli respectively).

### EEG signal analysis results

Next, we analysed the EEG data to identify the neural components that discriminated between congruent and incongruent trials. Specifically, for each participant separately, we performed a single-trial multivariate discriminant analysis to identify linear spatial weightings (i.e. spatial filters) of the EEG sensors that discriminated congruent from incongruent trials. The identified weightings produced a projection in the 128-dimensional EEG space that maximally discriminated congruent-vs-incongruent trials within short pre-defined windows of 50 ms, locked to stimulus onset.

Application of the resulting linear spatial filters to single-trial EEG data produces a measurement quantifying the discriminating component amplitude (*y*, see *Methods and materials*). These component amplitudes can be used as an index of the quality of categorizing the congruency of stimulus feature-response key mappings in each trial. In other words, higher amplitudes, negative or positive, indicate higher neural evidence for congruent or incongruent stimulus feature-response key mappings, while values closer to zero indicate less evidence of categorizing associative congruency.

To quantify the discriminator's performance over time, we used the area under a receiver operating characteristic curve (i.e. A_z_ value), coupled with a leave-one-trial-out cross validation approach, to control for overfitting. Compared to traditional approaches, which assume an A_z_ value of 0.5 as chance performance, we performed a permutation analysis using a leave-one-trial-out procedure that produced an A_z_ randomization distribution, to compute a group-average A_z_ value, that lead to a conventional significance level of *p* = 0.05.

**Fig. 3:**
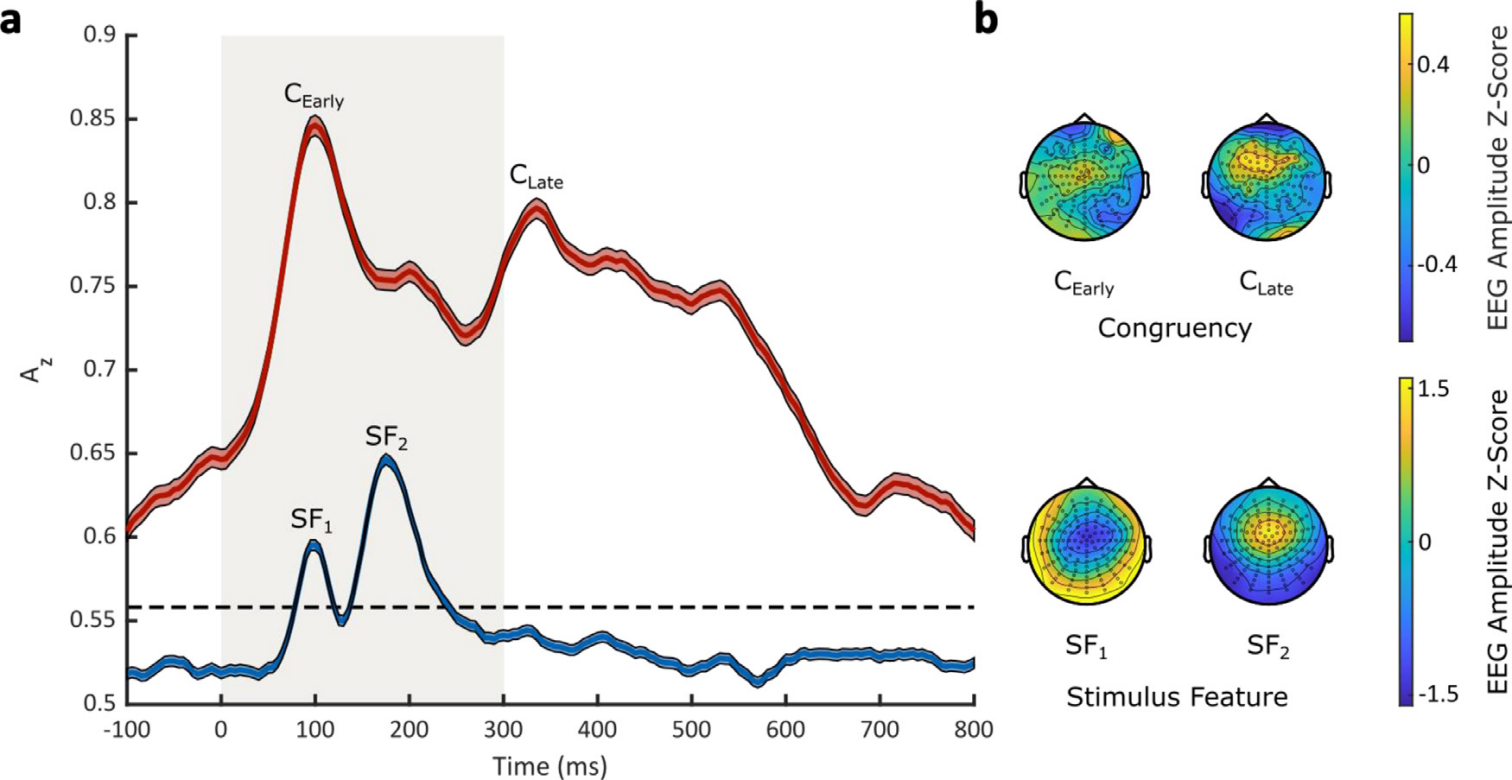
**Multivariate linear discriminant analysis results. a** Mean multivariate discriminator performance (A_z_), quantified by leave-one-out trial cross-validation procedure, during outcome discrimination of stimulus-locked EEG responses, as a function of congruency (congruent-vs-incongruent; red) and stimulus feature (high-pitch tones-vs-low-pitch tones; blue) conditions. Dashed black line represents the group average permutation threshold at *p* < 0.05 for congruent-vs-incongruent discriminator performance. Shaded error bars denote the standard error of the mean across participants. Shaded area denotes the presentation of auditory stimuli, from 0 ms (post-stimulus onset) to 300 ms (post-stimulus offset). **b** Scalp topographies at representative time windows corresponding to the two EEG components, defined for congruency (*Top*, C_Early_ and C_Late_) and stimulus feature (*Bottom*, SF_1_ and SF_2_) conditions respectively.

**Fig. 4:**
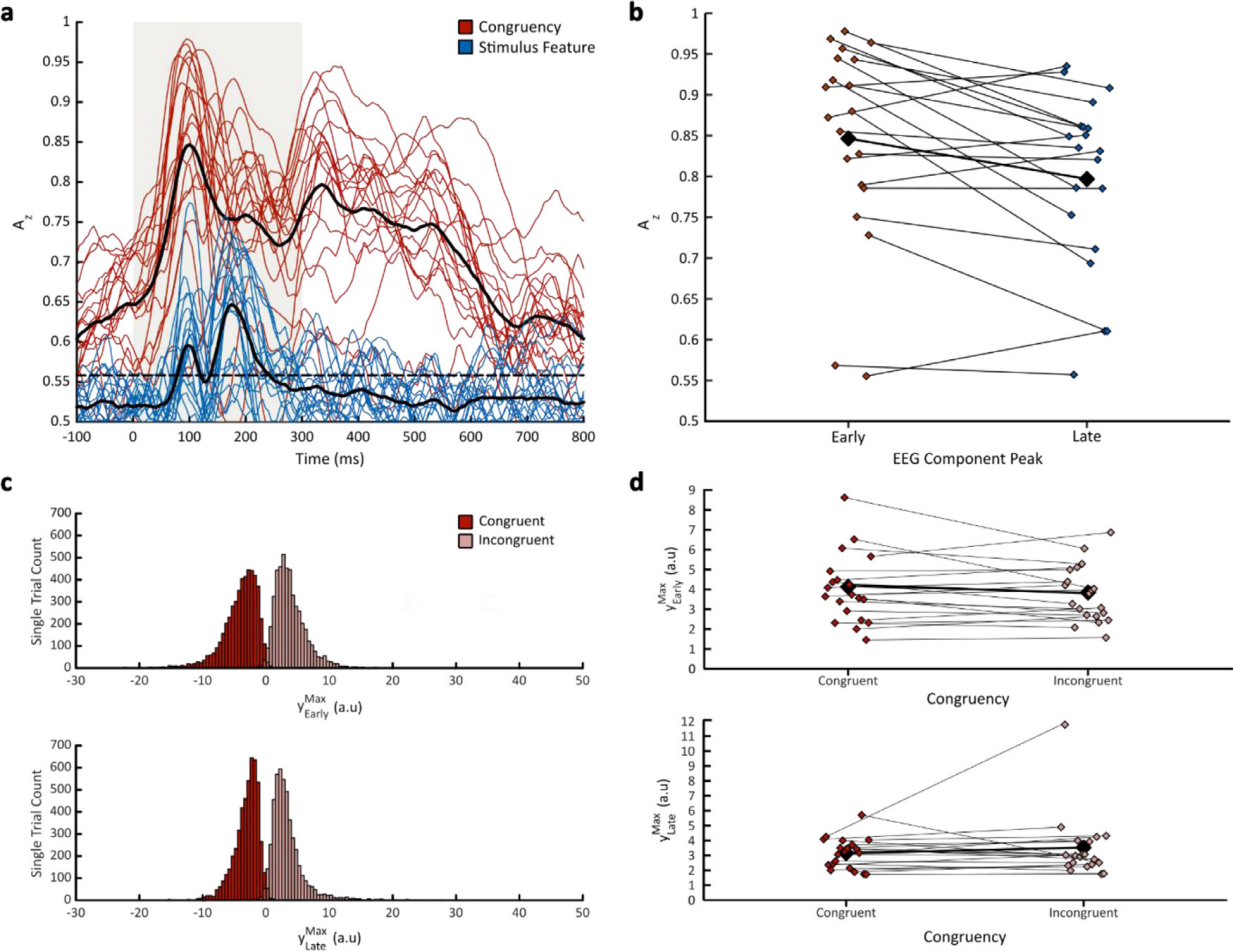
**Participant multivariate linear discriminant analysis results. a** Participants’ mean discriminator performance (A_z_), obtained from a leave-one-out trial cross-validation procedure, during stimulus feature (blue) and congruency (red) discrimination of stimulus-locked EEG responses. Dashed black line represents the group average permutation threshold at *p* < 0.05 for congruent-vs-incongruent discriminator performance. Condition mean discriminator performance (black) is also illustrated for congruency and stimulus feature discrimination**.** Shaded area denotes the presentation of auditory stimuli, from 0 ms (post-stimulus onset) to 300 ms (post-stimulus offset). **b** Participants’ mean discriminator performance (A_z_) for the Early and Late congruency-discriminating EEG components. **c** Single-trial discriminator amplitudes (y) for the Congruent (red) and Incongruent (pink) component amplitudes are illustrated as histograms for the Early (top) and Late (bottom) EEG components respectively. Negative values indicate neural evidence for congruency whereas positive values indicate neural evidence for incongruency. **d** Absolute values of our single-trial discriminator amplitudes (y) for the Congruent (red) and Incongruent (pink) component amplitudes for the Early (top) and Late (bottom) components respectively.

Our discriminator's performance as a function of stimulus-locked time revealed increased discriminant performance from 0 to 600 ms, above the significance level estimated from our permutation test. Specifically, discriminator performance within this range was characterized by two temporally specific components (Fig. 3a; C_Early_: mean peak time = 100–110 ms, Az value = 0.846; C_Late_: mean peak time = 330–340 ms, Az value = 0.797). These components were consistent across participants (see Fig. 4a for the A_z_ curves and Fig. 4b for the maximum A_z_ values of each participant). We then computed the corresponding scalp topographies, obtained using the forward model, correlating between peak discriminant output and EEG data (averaged over a 50 ms time window centred on the two classification performance peaks). For the ‘Early’ component, the strongest effects originated over central, left-lateralized centro-parietal, and left-lateralized occipital electrodes, whereas for the ‘Late’ component, the strongest effects predominantly originated over fronto-central electrodes. These results indicate that our multivariate LDA classifier identifies two EEG components that carry significant information about the congruency of stimulus feature-response key mappings.

Similarly, we applied the same single-trial multivariate discriminant analysis to the EEG data to identify the neural components that discriminated between trials that presented high-pitch and low-pitch auditory tones. Here, our discriminator's performance as a function of stimulus-locked time revealed increased discriminant performance post-stimulus onset, characterized by two temporally specific peaks (Fig. 3; SF_1_: mean peak time = 90–100 ms, Az value = 0.595; SF_2_: mean peak time = 170–180 ms, Az value = 0.647). The corresponding scalp topographies, again obtained using the forward model, revealed a bipolar EEG response that discriminated the two auditory stimuli. The first component (SF_1_) had positive activations over outer occipital, parietal, and temporal electrodes and negative activations over a frontocentral cluster, whereas the second component (SF_2_) showed activations at the same locations with inverse polarity. Notably, the stimulus-discriminating components occur approximately at the same temporal window as the Early congruency-discriminating EEG component. Thus, taken together, our EEG results attribute an early sensory-encoding role for the Early congruency-discriminating component and a post-sensory role for the Late congruency-discriminating component.

### Neurally-informed cognitive modelling results

**Fig. 5:**
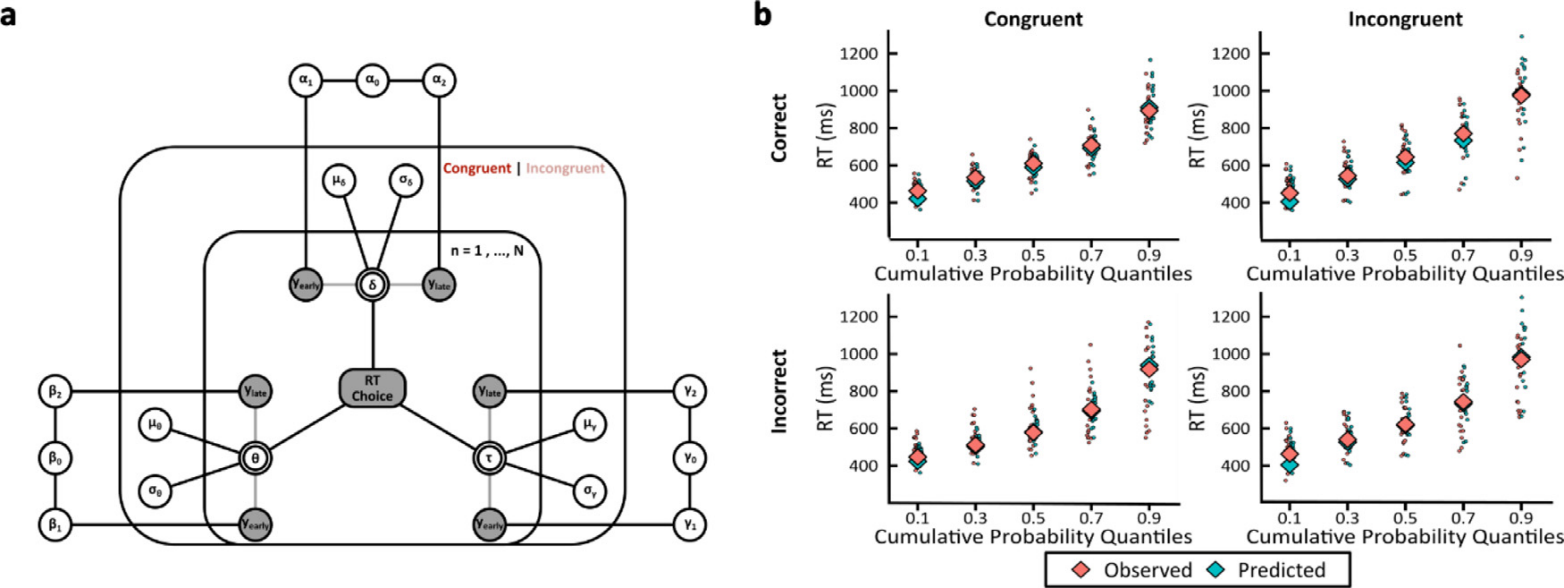
**Neurally-informed cognitive modelling. a** Graphical representation illustrating the Bayesian hierarchical framework for estimating neurally-informed HDDM parameters. Round nodes represent continuous random variables, with shaded nodes representing recorded or computed signals, i.e., single-trial behavioural data (RTs and Choice) and EEG component discriminator amplitudes (y's). Double-bordered nodes represent deterministic variables, defined in terms of other variables. Plates denote a hierarchical framework for modelling multiple random variables. The inner plate is over participants (*n* = 1, …, N) and the outer plate is over congruency conditions (Congruent | Incongruent). Parameters are modelled as random variables with inferred means µ and variances σ^2^, constrained by inferred estimates over congruency conditions. External plates denote constructed single-trial regression coefficients as predictors of the drift rate (ɑ), decision boundary (θ), and non-decision time (τ). **b** Posterior predictive checks of the Neurally-informed HDDM fitting to participant and group behavioural data. Modelling fit to behavioural data was assessed using a cumulative quantile-probability plot, showing quantiles of RT distributions split across congruency conditions (Congruent/Incongruent in columns) and choice accuracy (Correct/Incorrect in rows). Cumulative probability quantiles are plotted along the x-axis for observed RTs (in pink), i.e. single-trial behavioural data (RTs), and predicted RTs (in cyan), i.e. simulated RTs from HDDM posterior predictive estimates. Diamonds represent group averages and circles represent single-participant values.

After characterizing the effect of congruency on the discriminating power of brain activity, we sought to gain a mechanistic insight into how the identified single-trial neural responses were linked to improvements in perceptual decision formation between congruent and incongruent trials. To achieve this, we used a neurally-informed variant of the Hierarchical Drift Diffusion Model (HDDM; Wiecki et al., 2013, see Fig. 5a for a graphical illustration and *Methods and materials* for details on the model). As previously mentioned, the HDDM is a Bayesian implementation of the well-known Drift Diffusion Model, used for characterizing perceptual decision formation in 2AFC paradigms (DDM; Ratcliff and McKoon, 2008).

We extracted the maximum single-trial discriminator amplitudes ($|y_{early}^{max}|$ and $|y_{late}^{max}|$) from subject-specific temporal windows corresponding to our stimulus-locked ‘Early’ and ‘Late’ peak EEG components. These values represent the neural evidence for discriminating the congruency of stimulus feature-response key mappings per trial (see Fig. 4c for histograms of $y_{early}^{max}\;$and $y_{late}^{max}$ in congruent and incongruent trials). Depending on the stimulus feature-response key mapping, these values demonstrate where stimulus-induced neural responses systematically differ, explicitly linking perceptual decision formation benefits to time periods where early bottom-up and late top-down influences from associative congruency modulate the subsequent neural responses. Thus, we used them to construct regressors for drift rate, boundary separation, and non-decision time parameters in the model. We estimated regression coefficients to assess the relationship between trial-to-trial variations in EEG component amplitude and parameter posterior estimations (Coefficients $\alpha_{1},\;\beta_{1},\;\gamma_{1}$ and $\alpha_{2},\;\beta_{2},\;\gamma_{2}$ for $|y_{early}^{max}|$ and $|y_{late}^{max}|$ respectively). Note that we extracted the *absolute* single-trial discriminator amplitudes, as this would permit us to compare indexes of neural evidence, underlying our assumption that larger component amplitudes reflect higher discriminant activity within the brain for congruent compared to incongruent trials (see Fig. 4d for the average $|y_{early}^{max}|$ and $|y_{late}^{max}|$ of each participant).

**Fig. 6:**
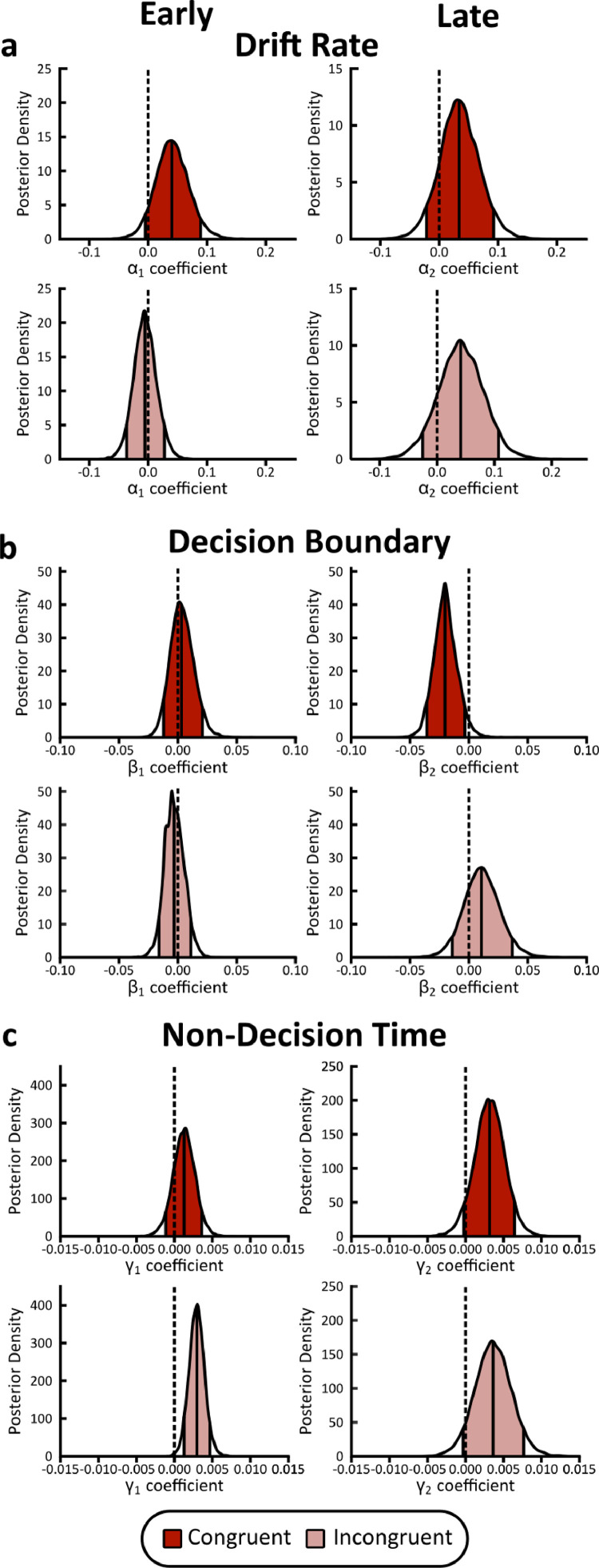
**Neurally-informed cognitive modelling results**. Posterior density distributions of estimated regression coefficients for **a** drift rate (α’s), **b** decision boundary (β’s), and **c** non-decision time (γ’s) for Early (Left) and Late (Right) EEG component discriminator amplitudes. All regression coefficients are derived from the neurally-informed HDDM, including *N* = 20 independent participants and 9850 trials. Thick lines denote the median point estimate and the shaded areas represent the 90% probability mass, enclosed between 5% and 95% probability confidence intervals. Dashed lines denote the zero point.

We found a good fit of the behavioural data (i.e. choice accuracy and RTs) from our proposed neurally-informed HDDM (Fig. 5b). Crucially, we found that the single-trial amplitudes for the Early component were highly predictive of increases in non-decision time estimates for incongruent trials (Early: $P(\gamma_{1}^{Congruent}$ < 0) = 0.189, log-odds = −1.470; $P(\gamma_{1}^{Incongruent}$ > 0) = 0.997, log-odds = 5.861. Late: $P(\gamma_{2}^{Congruent}$ < 0) = 0.62, log-odds = −2.712; $P(\gamma_{2}^{Incongruent}$ > 0) = 0.936; log odds = 2.690; Fig. 6c). We should note that the non-decision time parameter captures the duration of non-decisional processes, such as the latency of early stimulus encoding and the motor preparatory response. This result is consistent with the longer RTs observed in incongruent trials, and combined with the early occurrence of this component (∼100 ms post-stimulus onset), suggests a longer duration of early sensory processing during incongruent trials.

We further found evidence to indicate that single-trial amplitudes of the Late component were highly predictive of decreases in decision boundary parameter estimates for congruent trials only (Early: P($\beta_{1}^{Congruent}$ < 0) = 0.370, log-odds = −0.553; P($\beta_{1}^{Incongruent}$ < 0) = 0.641, log-odds = 0.580. Late: $P(\beta_{2}^{Congruent}$ < 0) = 0.973, log-odds=3.574; P($\beta_{2}^{Incongruent}$ < 0) = 0.234, log-odds = −1186; Fig. 6b). Thus, this implies a modulation of the decision boundary in congruent trials by the Late component amplitudes. The lower decision boundary indicates that participants require less evidence to reach a decision in congruent trials, thus they a) respond faster and b) are more likely to make incorrect perceptual judgments when responding fast. These are consistent with our behavioural findings indicating a) shorter RTs in congruent trials and b) faster RTs for incorrect choices compared to correct choices in congruent trials (Fig. 2).

## Discussion

In this work, we used single-trial multivariate linear discriminant analysis and neurally-informed cognitive modelling to investigate the neural mechanisms underlying auditory pitch-visual size cross-modal associations, formulated from the presentation of unisensory stimulus features (i.e. auditory pitch). Using a variant of the Implicit Association Test (Parise and Spence, 2012), we showed significant behavioural improvements as a result of associative congruency as participants responded faster to congruent than incongruent stimulus feature-response key mappings (Fig. 2). Our multivariate linear discriminant analysis on the EEG signals revealed neural information for congruent mappings in a 0–600 ms post-stimulus onset window. Moreover, we characterised two EEG components carrying congruency-relevant information in single trials: an ‘Early’ (∼100–110 ms) component and a ‘Late’ (∼330–340 ms) component. Using neurally-informed cognitive modelling, we linked these neural correlates of associative congruency with the corresponding behavioural benefits for forming perceptual decisions. We thus associated the observed shorter RTs in congruent trials with a) an increase in the duration of sensory processing time modulated by the Early component during incongruent trials, and b) a decrease in the quantity of post-sensory evidence needed to facilitate a perceptual choice modulated by the Late component in congruent trials.

Our behavioural results provide further evidence supporting the existence of auditory pitch-visual size cross-modal associations that have been previously reported (Bien et al., 2012; Evans and Treisman, 2010; Gallace and Spence, 2006; Parise and Spence, 2009; 2008; 2012). More importantly, our results demonstrate that auditory pitch-visual size cross-modal associations can be formulated even when only a single unisensory stimulus feature is presented on a single-trial basis. This replicates the findings of Parise and Spence (2012), who reported faster RTs for congruent compared to incongruent trials for five auditory-visual stimulus combinations, including frequency-pitch and object-size. It should be emphasized that the benefits of associative congruency observed in our study should be considered relative in nature. Specifically, it is the variation within blocks of trials, and subsequent trial-by-trial contrasts between ‘high’ and ‘low’ pitch tones, that influences behavioural performance, and not necessarily the absolute pitch frequency of the auditory tones presented (Spence, 2019).

We further provide neuroimaging evidence demonstrating a robust modulation to neural activity by the associative congruency of auditory-driven stimulus feature-response key mappings. Importantly, as the IAT only presents one unisensory stimulus feature per trial, it minimizes modulations from confounding neural activity attributed to further multisensory decision-making mechanisms, notably multisensory integration (Franzen et al., 2020; Mercier and Cappe, 2020) and a form of selective-attention/attention-dividing (Bien et al., 2012, Marks, 2004) between two simultaneously presented stimulus features.

To examine neural activity specifically related to the behavioural benefits of cross-modal associative congruency, we applied multivariate Linear Discriminant Analysis to decode congruent from incongruent stimulus-feature response key mapping trials. The application of multivariate Linear Discriminant Analysis to our EEG data revealed two temporally distinct neural components representing both early and late influences of associative congruency mappings. Furthermore, the two components share a broadly consistent scalp topography for localizing associative congruency benefits, clustering a positive discriminative topography that emerged over left-lateralized centro-parietal, and left-lateralized occipital electrodes, gradually emerging toward fronto-central regions of the brain.

The first component (Early: ∼100–110 ms) arises near simultaneously with the defined components for encoding auditory stimuli (i.e. SF_1_ and SF_2_), with higher neural evidence for discriminating associative congruency prior to discriminating auditory pitch. The early latency onset of the discrimination of congruency coincides with our results revealing an increase in discrimination of the presented auditory stimulus feature, possibly implicating an overlapping mapping of perceptual priors of auditory-driven pitch-size associations that automatically influences early sensory encoding/processing. We suggest that the benefits of associative congruency, observed in the behavioural results, modulate neural activity due to a form of perceptual feedback, influencing the early processing of sensory information across the different modalities during the perceptual decision formation process. Previous research has demonstrated that repeated exposure to complementary stimulus features shapes their multisensory composition, thus forming implicit preferences to congruent mappings (Habets et al., 2017; Kayser and Kayser, 2018; Park and Kayser, 2019). Therefore, this reaffirms our assumption that associative congruency shapes multisensory decision formation, thus improving either or both the speed and accuracy of choice. Similarly, multisensory enhancements during perceptual decision formation have been found with interactions occurring in neural signals at very short latencies (Boyle et al., 2017; Cappe et al., 2010; Foxe et al., 2000; 2002; Foxe and Schroeder, 2005; Molholm et al., 2002; 2006; Sperdin et al., 2009). Importantly, in our study the early modulation we observed suggests such enhancements are not exclusively multisensory, since on each trial only a single sensory stimulus was presented. Consequently, we contend that the early onset of our results suggests that cross-modal associations are not exclusively decision-related, but may be perceptual in origin.

Alternatively, an existing underlying mapping of the perceptual priors of auditory pitch-visual size associations may automatically influence early sensory encoding. Cross-modal associations reflect a naturally occurring mapping between stimulus features (Parise et al., 2014; Parise and Spence, 2013). Auditory acoustic pitch-visual size associations demonstrate a strong statistical correspondence in our external environment, whereby larger objects resonate at lower pitch frequencies than smaller objects. Thus, an alternative interpretation suggests that the early onset of our results is related to the influence of such existing priors shaped in the statistics of our natural environment (Baier et al., 2006). For example, if top-down processes access this existing mapping, and signal to early sensory encoding regions, such feedback might embed the existing environmental prior mapping. The contention of an influence of existing priors between auditory pitch and visual size further contributes to the longstanding debate in the field concerning the degree of automaticity of cross-modal associations (Chen and Spence, 2017; Spence and Deroy, 2013). Our interpretation here supports findings suggesting that the automaticity of audiovisual associative congruency benefits involves both perceptual bottom-up and modulatory top-down processes (Getz and Kubovy, 2018). This interpretation is further supported by the observation that our discriminator's performance for congruency exceeded the significance level prior to auditory stimulus feature presentation (i.e. Az > 0.05, see Fig. 3). A possible explanation for this is that the discrimination of EEG component amplitudes, formulated by the congruency of stimulus feature-response key mappings prior to the formation of perceptual decisions, could indicate pre-mapping anticipation, or expectation, that actively modulates the effects of congruency benefitting the faster formation of perceptual decisions, without modulating the categorisation of auditory stimulus features, or their sensory signals themselves. Bang and Rahnev (2017) present psychophysical evidence to implicate the effects of pre-stimulus anticipation to support this interpretation.

Thus, we contend that cross-modal associations may benefit from being consolidated within a predictive coding framework as a mechanism benefitting choices for multisensory decision-making (Shi and Burr, 2016; Talsma, 2015). In this framework, repeated exposure to auditory pitch-visual size mappings could relate to some existing underlying mapping of the perceptual priors between high/low-pitch tones, and small/large-size objects respectively. In a predictive coding framework, we posit that the early sensory benefits we observed from associative congruency may be influenced by newly formed priors of auditory pitch-visual size associations, with top-down processing signalling to early sensory regions of the brain providing feedback that embeds this environmental prior. Evidence that applies a predictive coding framework stems from studies that implement Bayesian interpretations of the effect of existing priors (Huang and Rao, 2011; McGovern et al., 2016; Tong et al., 2020). Bayesian theories have implicated that cross-modal associative congruency strengthens the binding of stimulus features during multisensory integration (Parise and Spence, 2013), demonstrating the pronounced effect of associative priors for benefitting multisensory decision formation (Acerbi et al., 2018; Gau and Noppeney, 2016; Rohe et al., 2019; Rohe and Noppeney, 2015a; 2015b).

The late onset of the second component (Late: ∼330–340 ms) further suggests that cross-modal associations may be decision-related, albeit not exclusively. Previous perceptual decision formation studies have consolidated a neural signature of decision formation, often termed Centro-Parietal Positivity (CPP; O'Connell et al., 2018; Polich, 2007; Tagliabue et al., 2019; Twomey et al., 2016), or the late decision-related component (Philiastides et al., 2006, 2011; 2014; Philiastides and Sajda, 2006; 2007), arising approximately 300–500 ms post-stimulus, reflecting neural activity for accumulating evidence to facilitate a choice. A previous study by Mercier and Cappe (2020) has further attributed that the CPP indexes the accumulation of sensory evidence for multisensory decision-making. In our study, the decoded Late component highly resembles the spatiotemporal characteristics of this indexed neural signature, with a positive discriminative topography emerging across centro-parietal regions. Given we further observed higher neural evidence for discriminating associative congruency as late as 600 ms, we contend that the congruency of cross-modal associations for accumulating sensory evidence at a further decisional stage is important, supporting studies demonstrating the CPP for both unisensory and multisensory decision-making, thus benefitting perceptual decision formation.

Previous multisensory decision-making studies have localized benefits to perceptual decision formation at a later stage (Franzen et al., 2020; Kayser et al., 2017). However, these cannot be solely attributed to congruency effects as previously explained. Here, by using the IAT, we were able to demonstrate that associative congruency has a further role in accumulating sensory evidence at a later decisional stage, with neural activity aligned with CPP, or the late decision-related component, even when a single unisensory stimulus feature is presented. Thus, we can localize the benefits of cross-modal associations for forming perceptual decisions while simultaneously minimizing the benefits that may be attributed to bottom-up (i.e. multisensory integration), or top-down (i.e. selective attention) multisensory processes.

When forming decisions with multisensory information, multisensory interactions are pervasive within the human brain, constituting different processes along the cortical hierarchy (Cao et al., 2019; Rohe et al., 2019; Rohe and Noppeney, 2016; Keil and Senkowski, 2018; Sadaghiani et al., 2009). For identifying when multisensory information benefits perceptual decision-making, three prominent theories persist in the field (Bizley et al., 2016): a) the early integration hypothesis, b) the late integration hypothesis, and c) what we term as the dual integration hypothesis, which was formulated by Mercier and Cappe (2020). The early integration hypothesis posits that early sensory encoding stages facilitate the influences of multisensory benefits from complementary sensory information across modalities (Ghazanfar and Schroeder, 2006; Kayser and Logothetis, 2007; Schroeder and Foxe, 2005). The late integration hypothesis, however, postulates that unisensory information is processed separately at early sensory encoding stages, then combined into a unified source of evidence at a late post-sensory decisional stage (Bizley et al., 2016; Franzen et al., 2020). Finally, the dual integration hypothesis posits that unisensory information is integrated at both early sensory encoding and later decision formation stages, consolidating a role of causal inference in determining whether multisensory information is supramodal in defining incoming sensory information (Aller and Noppeney, 2019; Cao et al., 2019; Gau and Noppeney, 2016; Kayser and Shams, 2015; Mercier and Cappe, 2020; Rohe et al., 2019; Rohe and Noppeney, 2015; Su, 2014).

Evidence supporting the early integration hypothesis arises from identified neural pathways between sensory cortices (i.e. the visual and auditory cortices), and higher-order associative cortices of the brain (e.g. parietal, temporal and frontal associative cortices), with cross-modal influences on neural responses localized early within the sensory cortices (Eckert et al., 2008; Ghazanfar and Schroeder, 2006; Giart et al., 1999; Kayser et al., 2017 Petro et al., 2017; Rohe and Noppeney, 2016). Previous research has also argued in favour of the late integration hypothesis, implicating post-sensory enhancements of decision evidence from a late integration of multisensory information benefits perceptual decision formation (Franzen et al., 2020). The processes of object recognition and categorization, naturally multisensory processes given the information presented to multiple sensory modalities, has led researchers to contend that top-down processing is required to determine associative congruency, thereby expediting the speed of perceptual decision formation. However, as we've previously discussed, evidence supporting the late integration hypothesis remains stemmed from paradigms that present two or more unisensory features simultaneously. By utilizing a paradigm that presents only one unisensory stimulus feature per trial, recorded neural activity elicits a neural component for discriminating stimulus feature-response key mapping congruency early in a trial. To implicate cross-modal associations are only post-sensory, or decisional, in origin ignores this early associative benefit for forming perceptual decisions and contradicts previous research localizing ERPs for associative congruency early in the decision-making process (Kovic et al., 2010; Bien et al., 2012). To briefly summarize, our results do not provide exclusive support for one of these two theories.

Our data do however support the dual integration hypothesis. Mercier and Cappe (2020) demonstrated support for this hypothesis in their study, in which they identified two temporally-distinct neural processes underlying multisensory decision-making across both cue detection and cue categorization paradigms. Importantly, decoding of EEG activity underlying unisensory signal cues implicated these processes were responsible for early sensory encoding and late decisional formation. Multisensory benefits observed in the behavioural data (i.e. faster RTs, higher accuracy, increased sensitivity towards multisensory cues) were concurrent with an acceleration of both processing stages, suggesting that associative congruency benefitted both a faster integration of sensory information and consolidation of decisional evidence. Here, we identified a similar temporal trajectory of EEG activity, characterized by two mechanisms complementing prior research demonstrating early sensory encoding and decision formation processes that benefit from cross-modal associative congruency (Bizley et al., 2016). Without confounds due to the processes of multisensory integration, and higher-order cognitive processes such as selective attention using the IAT, we also localized the effects of associative congruency as both early sensory-perceptual and late-decisional, thus further consolidating the benefits traditionally observed by early multisensory integrative processes and late decision accuracy. Ultimately, this is in line with a dual integration hypothesis, reconciling the early and late integration hypotheses respectively.

Importantly, our findings suggest that key mechanistic insights can be elicited by coupling models of perceptual decision formation with neuroimaging data. The inclusion of the two characterised EEG components enabled the disambiguation of the internal processes that yielded two IAT behavioural performance results. First, decreased RTs for congruent compared to incongruent stimulus feature-response key mappings, and second, decreased RTs for incorrect compared to correct congruent trials. Our Late component was linked with a decrease in the amount of evidence required to reach a decision as a result of congruent associations, thus congruent trials had shorter RTs and larger proportions of incorrect responses for short RTs. This result is complemented by the observation that incongruent stimulus-response mappings yielded increased non-decision time estimates modulated by the Early component, suggesting longer stimulus encoding times and consequently slower responses in incongruent trials.

Previous studies have used DDMs to study multisensory decision-making (Delis et al., 2018; Franzen et al., 2020; Kayser et al., 2017; Mercier and Cappe, 2020). To our knowledge, such studies have not focused purely on cross-modal associations and modelled behavioural and neuroimaging data from experimental paradigms that present two sensory stimuli simultaneously, or within close spatial or temporal proximity. The application of the IAT means we can model multisensory decision-making, yielding parameter estimates informed by neural measurements linked to the processing of one sensory stimulus feature, thus producing neurally compatible outcomes underlying benefits purely driven by cross-modal associations.

In conclusion, using a neurally-informed cognitive modelling approach, we first characterized the spatiotemporal dynamics of neural activity underlying associative congruency, and then probed its functional role in perceptual decision formation. By presenting only one unisensory stimulus feature per trial, we were able to overcome previous difficulties interpreting the mixed selectivity of neural responses to simultaneously presented stimulus features. Consequently, we could identify the effects of cross-modal associations on neural processing and draw a direct link between these neural processes and the behavioural benefits of associative congruency in perceptual decision-making. We recommend that future research consolidates our observations by utilizing similar unisensory approaches for investigating cross-modal associations with alternative statistical correspondences (e.g. auditory pitch-visual lightness; Brunel et al., 2015; Zeljko et al., 2019; auditory pitch-visual brightness; Marks, 1987; Klapetek et al., 2012; auditory pitch-visual elevation; Jamal et al., 2017; McCormick et al., 2018; Zeljko et al., 2019; auditory pitch-visual shape; Köhler, 1929; Marks, 1987; Parise and Spence, 2012, and higher-order semantic coherence; Marks, 2004; Parise and Spence, 2013; Revill et al., 2014; Sadaghiani et al., 2009; Spence and Deroy, 2013; Spence, 2011).

## CRediT author statement

**Joshua Bolam:** Conceptualization, Formal Analysis, Visualization, Writing – Initial Draft Preparation, Writing – Reviewing & Editing. **Stephanie C. Boyle:** Data Curation, Funding Acquisition, Investigation, Methodology. **Robin A.A Ince:** Data Curation, Investigation, Methodology, Validation **Ioannis Delis:** Conceptualization, Funding Acquisition, Resources, Supervision, Validation, Writing – Initial Draft Preparation, Writing – Review & Editing.

## Declaration of Competing Interest

The authors declare no competing interests.
